# Effect of Intra-articular Atelocollagen Injections for Patients With Knee Osteoarthritis: A Retrospective Chart Review

**DOI:** 10.7759/cureus.68954

**Published:** 2024-09-08

**Authors:** Man-Jun Park, Seung-Woo Ko, Jae-Ik Cho, Su-Hyun Lee, Hong-Kwan Shin

**Affiliations:** 1 Department of Orthopedic Surgery, Himnaera Hospital, Busan, KOR; 2 Department of Orthopedic Surgery, Madi Clinic, Seogwipo, KOR; 3 Department of Neurology, Cho Jae-ik Neurology, Seogwipo, KOR; 4 Department of Orthopedic Surgery, Lee Chun-Taek Hospital, Suwon, KOR; 5 Department of Orthopedic Surgery, Daegu Hanmi Hospital, Daegu, KOR

**Keywords:** intra-articular injection, pain management, knee osteoarthritis, chart review, injectable atelocollagen

## Abstract

Background

Osteoarthritis (OA) is a prevalent and exhausting condition often requiring long-term management. While there is a steady growth in the use of collagen-based treatment for OA, there is a lack of studies assessing the safety and efficacy of repeated administration of injectable atelocollagen for OA.

Objective

This study aims to evaluate the clinical efficacy and safety of repeated administration of injectable atelocollagen in reducing knee pain for patients with knee OA.

Methods

Clinical records of 91 patients from five hospitals were reviewed for this retrospective study. All 91 patients had received repeated administration of injectable atelocollagen (CartiPRO^®^, Dalim Tissen Co., Ltd., South Korea) as a treatment for knee OA for seven months. The efficacy of injectable atelocollagen was evaluated by physicians at least 30 days after the last administration, with outcomes categorized as “effective”, “moderately effective”, or “not effective”. For analysis purposes, both “effective” and “moderately effective” were grouped as “effective” while “not effective” was classified as “ineffective”. Safety was assessed by monitoring the incidence of adverse events (AEs) reported within six months following the re-administration of atelocollagen.

Results

Among the 91 patients, 96.7% (88 patients) experienced effective pain relief following the re-administration of CartiPRO^®^, with 3.3% (three patients) reporting ineffectiveness. In terms of safety assessment, 35 patients reported AEs, totaling up to 44 events, with no serious or unexpected device-related AEs.

Conclusion

The repeated use of atelocollagen was found to be both safe and effective in managing knee pain for patients with knee OA. These findings support the repeated use of injectable atelocollagen as a reliable treatment option for managing knee OA pain in clinical practice.

## Introduction

Osteoarthritis (OA) is one of the most common musculoskeletal disorders, with an estimated prevalence rate of 24% in the general adult population [1]. Due to the gradual rise of the aging population and an ongoing increase in obesity, OA has become a globally prevalent disease [2]. According to a study in 2023, 595 million people had OA in 2020, representing 7.6% of the global population. The knee was the most common site of OA with a projected estimate of a 74.9% increase in knee OA cases by the end of 2050 [3].

Knee OA, in particular, presents a significant burden due to its profound symptoms and chronic nature that pose a substantial impact on the patient’s daily lives. Knee OA, which is typically diagnosed at around the age of 55, often requires patients to endure the painful symptoms for several decades [4-6]. The impact of knee OA is further exacerbated by symptoms characterized by a reduced range of motion, muscle weakness, knee locking, swelling, and instability, all of which significantly impair daily activity and diminish the patient’s quality of life [7]. Given the significant challenges of knee OA on the functional independence and well-being of a patient, the validation for a safe and effective long-term treatment option is substantial.

The conventional treatment protocol for knee OA prioritizes the extensive use of conservative therapies by managing pain and maintaining natural joint integrity for as long as possible before switching to surgical procedures. Surgical interventions are only considered when all other options fail to manage the pain, which substantially impairs the patient’s quality of life [8].

Intra-articular drug delivery for the treatment of OA, compared with systemic administration, displays promising results, showing signs of reduced adverse events (AEs), faster onset of action, and convenient dosing intervals (infrequent as once every six months). However, over the past 20 years, intra-articular treatment options for managing OA have been limited to a few selections, such as analgesics, glucocorticoids, hyaluronic acid (HA), duloxetine, opioids, capsaicin, and topical analgesics, some of which lack clinical efficacy [9,10]. Currently, there are no treatment methods that could alter the progression of knee OA, with the primary goal of treatment being focused on pain management and functional improvement [11,12].

During the onset of OA, the acute inflammation on the knee often develops into chronic inflammation, leading to the exposure of chondrocytes in the joint cavity, changes in the osmotic pressure, and gradual migration of proteoglycans into the joint cavity, which disrupts the natural healing process [13,14]. Pro-inflammatory cytokines, such as tumor necrosis factor (TNF)-β, Interleukin (IL)-6, IL-1α and IL-1β are released, stimulating cartilage-degrading enzymes like a disintegrin and metalloprotease with thrombospondin motifs (ADAMTS) and matrix metalloprotease (MMP). Release of pro-inflammatory cytokines, such as TNF-β, IL-6, IL-1α, and IL-1β stimulates cartilage-degrading enzymes like ADAMTS and MMP [15]. Consequently, ADAMTS and MMP contribute to the degradation of extracellular matrix (ECM), including collagen [16]. Therefore, the administration of exogenous collagen has been explored as a potential alternative for OA therapy [17,18].

Collagen is a fundamental component of connective tissues and serves as a key structural protein in the human body, making up 45% to 75% of the dry weight of ligaments, tendons, and cartilage. Collagen, a critical element of the ECM, plays a vital role in supporting tissues and maintaining cellular structures [19]. Native collagen, when implanted in vivo, could trigger an immune reaction due to the presence of antigenic telopeptides on both ends of its molecule. However, the immunogenicity of native collagen can be significantly reduced by removing these telopeptides, resulting in the formation of atelocollagen, a form with significantly lower toxicity and immunogenicity. Because of these properties, atelocollagen is widely used in medical applications, including wound healing and cartilage substitution [20].

Given its importance, many studies have hypothesized that intra-articular injection of atelocollagen could alleviate pain by replenishing damaged collagen in patients with knee pain caused by OA or other cartilage defects. This hypothesis led to the development and clinical testing of various collagen-based intra-articular injections [21]. Despite growing interest in injectable collagen for treating OA, there is a lack of research on the safety and efficacy of repeated administration of atelocollagen in managing knee OA pain.

Therefore, we conducted a retrospective study to evaluate the safety and effectiveness of repeated administration of injectable atelocollagen in reducing and managing knee pain for OA patients.

## Materials and methods

Patients

In this multicenter retrospective study, clinical data of 91 patients who have been diagnosed with knee OA using the Kellgren-Lawrence (KL) grades I, II, and III, which were radiologically assessed, were collected for the analysis. The inclusion criteria for the clinical data were those who underwent re-administration of 180 mg of atelocollagen (CartiPRO®, Dalim Tissen Co., Ltd., South Korea) six months after the initial administration of 180 mg of atelocollagen (CartiPRO^®^). Clinical data with at least one month of medical records available, starting from May 2023 to September 2023, after the re-administration of CartiPRO^®^ were included in the study. Patients who received re-administration in different sites of the knee were excluded.

Data collection

Clinical data of 91 patients with a confirmed diagnosis of knee OA, with KL grades I, II, and III, were collected from five medical facilities, using the electronic medical record (EMR) system. The collected patient EMR data were analyzed and evaluated retrospectively.

Study variables (diagnostic criteria and clinical data)

The collected data include the basic patient information (initials, age, sex, date of birth, weight, and height), medical history, imaging data of patients with available magnetic resonance imaging and radiography findings, quantitative joint condition data evaluated by the KL grade, clinical assessments (physician’s assessment; rated as “effective,” “moderately effective,” or “ineffective”), medication history (use of analgesia within 28 days before and after re-administration), AEs, and concomitant medication history (medications administered together with CartiPRO^®^).

Clinical assessment and safety evaluation

The clinical assessment was performed by the attending physicians at each hospital. The efficacy of re-administration of atelocollagen was evaluated with outcomes categorized as “effective”, “moderately effective”, or “not effective”, which are based on the degree of pain determined by palpation, patient questionnaire in the EMR, and the imaging data. Pain relief was determined based on the physician's assessment of pain addressed by patients and their subsequent use of medication. The absence of complaints regarding pain after re-administration of CartiPRO^®^ was considered “effective”, and a decrease in the severity of pain with reduced need for pain managing medication was considered “moderately effective”. Continued pain or the need for more medication in pain management was considered “not effective”. For analysis purposes, both “effective” and “moderately effective” were classified as “effective”, whereas “not effective” was classified as ineffective. EMR data on the AEs occurring within six months of CartiPRO^®^ re-administration were retrospectively reviewed and the results are presented in percentages. In addition to the clinical assessment, changes in the KL grades after CartiPRO^®^ re-administration were analyzed.

Statistical analysis

Differences in AE incidence by patient background and treatment factors were analyzed using the chi-square test. The percentages of each rating assigned by the physicians were calculated and analyzed. With the available data, changes in the radiological data and KL grade after administration of the product were analyzed using the chi-square test. Statistical analysis was performed using SAS^®^ software (Version 9.2, SAS Institute, Cary, North Carolina, US).

## Results

Demographics and clinical characteristics

Data of 91 patients who met the study criteria were collected in this study. The mean age of patients was 74.03 ± 10.07 years with 64.83% (59 out of 91) aged ≥70 years, 26.37% (24 out of 91) aged 60-69 years, and 6.59% (6 out of 91) aged 50-59 years. Overall, 82.42% (75 out of 91) of patients were elderly patients aged 65 years and older. The patients’ mean height, weight, and body mass index were 159.92 ± 7.35 cm, 63.02 ± 8.30 kg, and 24.58 ± 2.19 kg/m^2^, respectively (Table 1).

**Table 1:** Patients’ demographic and general characteristics UK, unknown; SD, standard deviation; BMI, body mass index

		Safety set ( n = 91)
Sex	Male	5 (5.49%)
	Female	85 (93.41%)
	UK	1(1.10%)
Age (years)	n	91
	Mean ± SD	74.03 ± 10.07
	Median (Min, Max)	75 (42, 95)
Age group (years)	19–29	0 (0.00%)
	30–39	0 (0.00%)
	40–49	2 (2.20%)
	50–59	6 (6.59%)
	60–69	24 (26.37%)
	≥70	59 (64.84%)
Older adult	<65	16 (17.58%)
	≥65	75 (82.42%)
Height (cm)	n	20
	Mean ± SD	159.92 ± 7.35
	Median (Min, Max)	159.50 (138.00, 170.00)
Body weight (kg)	n	20
	Mean ± SD	63.02 ± 8.30
	Median (Min, Max)	62.50 (50.00, 82.00)
BMI (kg/m^2^)	n	20
	Mean ± SD	24.58 ± 2.19
	Median (Min, Max)	24.61 (20.83, 29.30)

Medical history

Out of the collected data of 91 patients, 79.12% (72 patients) had a documented medical history. The medical histories of each patient were categorized using the latest version of the Medical Dictionary for Regulatory Activities (MedDRA; https://www.meddra.org/), as system organ class (SOC) and preferred term (PT).

According to the SOC classification, the most common medical history was "Musculoskeletal and connective tissue disorders" (61.54%; 56 out of 91), followed by "Metabolism and nutrition disorders" (27.47%; 25 out of 91), and both "Gastrointestinal disorders" and "Vascular disorders" (23.08%; 21 out of 91 in both). According to the PT classification, "Osteoporosis" was the most common condition (50.55%; 46 out of 91) followed by "Hyperlipidemia" (26.37%; 24 out of 91) and "Chronic gastritis," "Spinal osteoarthritis," and "Hypertension" (21.98%; 20 out of 91 each) (Table 2).

**Table 2:** Patients’ medical history SOC, system organ class; PT, preferred term

SOC	PT	Safety set (n = 91)
Blood and lymphatic system disorder		2 (2.20%)
	Anemia	2 (2.20%)
Cardiac disorder		4 (4.40%)
	Angina pectoris	2 (2.20%)
	Arrhythmia	1 (1.10%)
	Atrial fibrillation	1 (1.10%)
	Cardiac disorder	1 (1.10%)
Endocrine disorder		5 (5.49%)
	Hypothyroidism	5 (5.49%)
Gastrointestinal disorder		21 (23.08%)
	Chronic gastritis	20 (21.98%)
	Gastritis	1 (1.10%)
Hepatobiliary disorder		2 (2.20%)
	Hepatic steatosis	2 (2.20%)
Injury, poisoning, and procedural complication		3 (3.30%)
	Rib fracture	2 (2.20%)
	Spondylolysis	1 (1.10%)
Metabolism and nutrition disorder		25 (27.47%)
	Diabetes mellitus	10 (10.99%)
	Gout	4 (4.40%)
	Hyperlipidemia	24 (26.37%)
Musculoskeletal and connective tissue disorder		56 (61.54%)
	Back pain	1 (1.10%)
	Cervical spinal stenosis	1 (1.10%)
	Intervertebral disc disorder	2 (2.20%)
	Muscle disorder	1 (1.10%)
	Osteoporosis	46 (50.55%)
	Periarthritis	1 (1.10%)
	Rheumatoid arthritis	2 (2.20%)
	Spinal osteoarthritis	20 (21.98%)
	Spinal stenosis	4 (4.40%)
Nervous system disorder		11 (12.09%)
	Carpal tunnel syndrome	1 (1.10%)
	Cerebral infarction	1 (1.10%)
	Cognitive disorder	3 (3.30%)
	Dementia	6 (6.59%)
Psychiatric disorder		7 (7.69%)
	Anxiety disorder	3 (3.30%)
	Delusional disorder, unspecified type	1 (1.10%)
	Insomnia	3 (3.30%)
	Panic disorder	1 (1.10%)
	Sleep disorder	1 (1.10%)
Renal and urinary disorder		4 (4.40%)
	Glycosuria	1 (1.10%)
	Hematuria	3 (3.30%)
Respiratory, thoracic, and mediastinal disorder		1 (1.10%)
	Asthma	1 (1.10%)
Surgical and medical procedure		1 (1.10%)
	Bowel obstruction surgery	1 (1.10%)
Vascular disorder		21 (23.08%)
	Hypertension	20 (21.98%)
	Peripheral venous disease	2 (2.20%)
	Varicose vein	1 (1.10%)

Quantitative joint condition assessment

Quantitative joint condition assessment was measured by the KL grade. The mean KL grades before the re-administration of atelocollagen injection were 2.55 ± 0.88 and 2.55 ± 0.79 after re-administration. The mean changes were -0.03 ± 0.38. The changes in the KL grade before and after the re-administration of CartiPRO^®^ were not statistically significant (Table 3).

**Table 3:** Changes in the KL grade after re-administration of CartiPRO® a: Based on the latest data after administration, ￡: paired t-test, §: Wilcoxon signed-rank test SD, standard deviation; KL, Kellgren–Lawrence

	Efficacy set (n = 91)			
	Before administration	After administration^a^	Change (after administration–before administration)	p-value
n	84	77	70	
Mean ± SD	2.55 ± 0.88	2.55 ± 0.79	-0.03 ± 0.38	0.5310^￡^
Median (Min, Max)	3.00 (1.00, 4.00)	3.00 (1.00, 4.00)	0.00 (-2.00, 1.00)	0.7656^§^

Regarding changes in the KL grade after the re-administration of CartiPRO^®^, 1.43% of patients had the grade changed from G3 to G2 while 2.86% had the grade changed from G2 to G1 (Table 4). None of the patients with G4 or G1 at baseline had their grades lowered after re-administration.

**Table 4:** Changes in the KL grade after the re-administration of CartiPRO® ¢: McNemar's test

		Before administration					p-value
		G0	G1	G2	G3	G4	
After administration	Grade 0	0 (0.00%)	0 (0.00%)	0 (0.00%)	0 (0.00%)	0 (0.00%)	0.9477^¢^
	Grade 1	0 (0.00%)	6 (8.57%)	2 (2.86%)	0 (0.00%)	0 (0.00%)	
	Grade 2	0 (0.00%)	1 (1.43%)	20 (28.57%)	1 (1.43%)	0 (0.00%)	
	Grade 3	0 (0.00%)	1 (1.43%)	2 (2.86%)	31 (44.29%)	0 (0.00%)	
	Grade 4	0 (0.00%)	0 (0.00%)	0 (0.00%)	0 (0.00%)	6 (8.57%)	

Clinical assessment

Treatment with CartiPRO® was found to be effective in 73.63% of patients (67 out of 91), moderately effective in 23.08% (21 out of 91), and not effective in 3.30% (3 out of 91).

In evaluating the efficacy, “effective” and “moderately effective” were categorized as effective, whereas “not effective” was categorized as ineffective. After the introduction of CartiPRO^®^, 96.70% (88 out of 91) cases were considered effective and 3.30% (3 out of 91) cases were considered ineffective.

Safety evaluation

Among the clinical data of 91 patients, 38.46% (35 out of 91, total of 44 events) developed an AEs. However, there were no device-related AEs, serious adverse events (SAEs), serious device-related AEs, unexpected AEs, unexpected device-related AEs, serious and unexpected AEs, or serious and unexpected device-related AEs (Table 5).

**Table 5:** Incidence of AEs by type a: All AEs excluding those that are “not related” or “likely not related” to the medical device AE, adverse event; SAE, serious adverse event; CI, confidence interval

	Safety set (n = 91)		
	n (%)	95% CI	Number of events
AEs	35 (38.46%)	(28.45%, 49.25%)	44
Device-related AE^a^	0 (0.00%)	0 (0.00%)	0
SAE	0 (0.00%)	0 (0.00%)	0
Medical device-related SAE	0 (0.00%)	0 (0.00%)	0
Unexpected AE	0 (0.00%)	0 (0.00%)	0
Unexpected device-related AE	0 (0.00%)	0 (0.00%)	0
Serious and unexpected AE	0 (0.00%)	0 (0.00%)	0
Serious and unexpected device-related AE	0 (0.00%)	0 (0.00%)	0

Regarding the incidence of AEs according to the use of analgesics, AEs occurred in 48.98% (24 out of 49 patients, 29 events) among patients who used analgesics and 26.19% (11 out of 42 patients, 15 events) among patients who did not use analgesics.

Concomitant medication history

In this study, 72.53% of patients (66 out of 91) used concomitant medications. These medications were classified according to the anatomical therapeutic chemical (ATC) code level 1 (anatomical main group) and level 2 (therapeutic subgroup). According to the ATC code level 1 classification, medications for the "MUSCULOSKELETAL SYSTEM" were the most frequently administered (31.87%; 29 out of 91), followed by drugs for the "ALIMENTARY TRACT AND METABOLISM" (30.77%; 28 out of 91), "NERVOUS SYSTEM" (18.68%; 17 out of 91), and "CARDIOVASCULAR SYSTEM" (17.58%; 16 out of 91) (Table 6).

**Table 6:** Concomitant drugs †: duplicate counting, ATC, anatomical therapeutic chemical

ATC level 1†	ATC level 2†	Safety set n = 91
Alimentary tract and metabolism		28 (30.77%)
	Antiemetics and antinauseants	1 (1.10%)
	Bile and liver therapy	1 (1.10%)
	Drugs for acid-related disorders	20 (21.98%)
	Drugs for functional gastrointestinal disorders	1 (1.10%)
	Drugs used in diabetes	4 (4.40%)
	Mineral supplements	5 (5.49%)
	Vitamins	1 (1.10%)
Anti-infectives for systemic use		1 (1.10%)
	Antivirals for systemic use	1 (1.10%)
Blood and blood-forming organs		11 (12.09%)
	Antianemic preparations	6 (6.59%)
	Antithrombotic agents	7 (7.69%)
	Blood substitutes and perfusion solutions	1 (1.10%)
Cardiovascular system		16 (17.58%)
	Agents acting on the renin-angiotensin system	6 (6.59%)
	Calcium channel blockers	3 (3.30%)
	Cardiac therapy	1 (1.10%)
	Lipid-modifying agents	8 (8.79%)
	Peripheral vasodilators	2 (2.20%)
	Vasoprotectives	3 (3.30%)
Dermatologicals		3 (3.30%)
	Antibiotics and chemotherapeutics for dermatological use	1 (1.10%)
	Antifungals for dermatological use	1 (1.10%)
	Corticosteroids, dermatological preparations	1 (1.10%)
Musculoskeletal system		29 (31.87%)
	Anti-inflammatory and antirheumatic products	15 (16.48%)
	Muscle relaxants	11 (12.09%)
	Other drugs for disorders of the musculoskeletal system	6 (6.59%)
Nervous system		17 (18.68%)
	Analgesics	2 (2.20%)
	Anesthetics	5 (5.49%)
	Other nervous system drugs	2 (2.20%)
	Psychoanaleptics	6 (6.59%)
	Psycholeptics	5 (5.49%)
Respiratory system		3 (3.30%)
	Cough and cold preparations	2 (2.20%)
	Drugs for obstructive airway diseases	1 (1.10%)
Systemic hormonal preparations, excluding sex hormones and insulins		8 (8.79%)
	Corticosteroids for systemic use	8 (8.79%)

## Discussion

OA is a prevalent degenerative joint disease that affects a significant number of the global population. Knee OA, which is the most common form of OA, severely impacts the patient's quality of life through chronic pain and reduced mobility, often requiring long-term management. Traditional treatments focus on pain management and maintaining joint function, with surgical interventions reserved as a final option. Although intra-articular drug delivery shows promising results for OA treatment, options are limited, with no existing therapies that improve the disease's progression. Collagen, a key structural protein in cartilage, has been explored for its potential to relieve OA symptoms through intra-articular injections. With the growing interest regarding the use of collagen to manage OA pain, multiple studies have been conducted in hopes of finding an effective therapeutic option [21]. However, due to the limited amount of information regarding the safety and effectiveness of repeated collagen injections, this study was conducted to evaluate the efficacy of atelocollagen re-administration for knee OA pain management.

In this study, we evaluated the safety and efficacy of knee OA treatment during a minimum follow-up period of seven months. A minimum of one month after the last administration of CartiPRO®, the treatments were reported as 'effective' in 96.70% of patients and 'ineffective' in 3.30%, confirming its efficacy in alleviating knee pain in patients with OA.

Different studies found that both HA and atelocollagen demonstrate similar clinical effectiveness in managing knee OA symptoms [1,22]. With similar safety and efficacy, the use of atelocollagen is considered a more efficient option than HA for patients [22]. In this study, 96.70% of patients reported a reduction in OA-related knee pain following atelocollagen treatment. Although the absence of a control group in our study limits the ability to draw definitive conclusions, these findings suggest that atelocollagen effectively manages and reduces OA-related knee pain. Future research should compare these findings with studies that include control or placebo groups, particularly to contextualize the efficacy and safety profiles of atelocollagen.

The exact biochemical mechanism involved in the pharmacological anti-arthritic effects induced by collagen is not clearly established [23]. However, collagen demonstrates excellent biocompatibility, negligible immunogenicity, specific interactions with growth factors and cell adhesion molecules, and biodegradability, as well as allowing inward cell growth and remodeling [24]. Collagen has been used as an ideal scaffold for cartilage tissue engineering and many studies have shown that collagen hydrogels are the most prospective material for cartilage regeneration [25]. Moreover, the specific acid sequence in the collagen structure provides numerous adhesion sites for cells, facilitating the delivery of chondrocytes or stem cells for chondrocyte derivatization and ECM formation [26].

According to the study conducted by Kim et al. (2020), type I atelocollagen exhibits no immunogenicity while significantly enhancing the chondrogenic biocompatibility and differentiation of hMSCs, which are indicated by elevated expression of chondrogenic markers such as Sox9, aggrecan, and type II collagen [27]. These findings suggest that type I atelocollagen offers a supportive three-dimensional structure that promotes cell condensation and differentiation, highlighting its potential for cartilage tissue regeneration.

Collagen, especially types I and II, plays a critical role in cartilage regeneration by influencing chondrocyte behavior and ECM formation. Collagen interacts with integrins on chondrocyte surfaces, activating signaling pathways that promote chondrogenic differentiation and ECM production. Additionally, type I collagen enhances cell condensation, a vital step in chondrogenesis, by upregulating adhesion molecules like N-Cadherin and N-CAM, which are essential for forming a functional cartilage matrix [27]. Moreover, collagen's role in balancing chondrogenic and hypertrophic differentiation is crucial for long-term cartilage stability. Type I collagen scaffolds have been shown to reduce the expression of hypertrophic markers, such as Runx2 and MMP13, potentially by maintaining Sox9 expression, which inhibits chondrocyte maturation and ossification [27,28].

Out of the 91 collected patient medical records, 38.46% reported AEs. However, there were no events of device-related AEs, unexpected AEs, or SAEs. Regarding the incidence of AEs in patients who used analgesics, the incidence of AEs was 48.98% among patients who used analgesics and 26.19% among patients who did not use analgesics.

Changes in the KL grades were examined for analysis purposes. There were no significant changes in the KL grade after the re-administration of CartiPRO^®^ compared to the baseline. Given that this study retrospectively collected the degree of pain relief in patients, we could not compare the changes using imaging data at particular time periods before and after the re-administration of the CartiPRO^®^. This indicates that further research is needed to investigate the effects of intra-articular injections of atelocollagen on cartilage regeneration. Patient data with KL grade 4 were excluded from data collection because advanced stages of knee OA typically require surgical interventions, such as arthroplasty or osteotomy, as definitive treatments [29].

The primary objective for treating knee OA is to prevent the need for total knee replacement in patients with severe cartilage damage. Clinical interventions in the early stages of OA are crucial for preserving joint on and delaying disease progression. While the use of HA injections to manage OA-related pain is commonly used, their effectiveness in preventing continuous cartilage degeneration remains uncertain. As such, intra-articular atelocollagen injections may serve as a more suitable intervention for early-stage OA patients. Patient screening is essential to minimize adverse reactions and optimize atelocollagen treatment. Atelocollagen treatment should be avoided in patients with known allergies to porcine proteins and chronic knee inflammations, such as rheumatoid arthritis. Additionally, the use of ultrasound-guided injections is recommended to ensure precise delivery into the knee joint [30].

While our study offers valuable insights, it is essential to acknowledge several limitations that may affect the interpretation of the findings. The small sample size and absence of a control group limit the generalizability of the findings, as we cannot definitively attribute pain relief to atelocollagen injections without baseline comparisons. Additionally, the reliance on physician assessments, rather than standardized tools like the Visual Analog Scale (VAS) or Western Ontario and McMaster Universities Osteoarthritis Index (WOMAC), introduces potential bias, as subjective assessments may vary between physicians and lack consistency. The retrospective design and the reliance of our data solely on the EMR without follow-up visits restricts the evaluation of pain and AEs to recorded EMR data.

To overcome the limitations, future research should focus on larger, controlled studies, ideally randomized trials, to better assess the safety and efficacy of atelocollagen injections. Using standardized pain scales would minimize bias, and follow-up visits could improve safety monitoring. Long-term studies are needed to assess the need for repeated injections and evaluate lasting benefits, with advanced imaging providing insights into cartilage regeneration.

## Conclusions

This study confirmed the use of atelocollagen injection (CartiPRO^®^) in alleviating knee pain. The safety of CartiPRO^®^ was also established, as evidenced by the absence of device-related AEs, unexpected AEs, and SAEs. These findings support the repeated use of intra-articular atelocollagen injection as a reliable and safe treatment option for managing knee OA in clinical practice. The present study provides a foundation for future research on repeated pain relief using atelocollagen in patients with knee OA. Furthermore, it contributes to the development of clinical approaches by evaluating the safety and efficacy of second-round administration of intra-articular injections of atelocollagen products. Further prospective research, including randomized clinical trials, should be conducted in larger populations.
